# Design of a lateral flow assay targeting the conserved NIID_2019-nCoV_N gene region for molecular viral diagnosis

**DOI:** 10.1590/1414-431X2025e14761

**Published:** 2025-10-13

**Authors:** D. Çam Derin, E. Gültekin

**Affiliations:** 1 Department of Molecular Biology and Genetics, Inonu University, Malatya, Turkey

**Keywords:** Lateral flow assay, Molecular diagnosis, NIID_2019-nCOV_N, SARS-CoV-2, Rapid assay

## Abstract

SARS-CoV-2, the causative agent of the COVID-19 pandemic, remains a significant threat to global public health. Therefore, rapid and accurate detection of the virus continues to be of critical importance. Among the specific gene regions of SARS-CoV-2, the Nucleocapsid (N) protein gene is one of the most frequently targeted for viral identification, with NIID_2019-nCOV_N being a notable example. While reverse transcriptase polymerase chain reaction (RT-PCR) remains the gold standard for diagnosis, alternative molecular detection methods are still limited. In this study, a lateral flow assay (LFA) was developed for the detection of a conserved gene region within NIID_2019-nCOV_N. Gold nanoparticles (AuNPs) were employed to enable visual detection, and the assay was designed based on nucleic acid hybridization principles. Two different membrane types (M17 and M12), three oligonucleotide probe concentrations (2, 4, and 8 µM) conjugated to AuNPs, and the assay's limit of detection (LOD) were evaluated. The target sequence from NIID_2019-nCOV_N was successfully detected by the naked eye within 5-6 min. No significant differences in performance were observed between the two membrane types across all probe concentrations, and the LOD was determined to be 1 pM. Consequently, the nucleic acid-based lateral flow assay (NABLFA) designed in this study, which targets a specific conserved base sequence, demonstrated high potential for rapid and sensitive molecular detection of SARS-CoV-2. Furthermore, this approach may be adapted for the identification of emerging viral variants or future outbreaks.

## Introduction

Coronaviruses have caused three major epidemics over the past two decades: severe acute respiratory syndrome (SARS), Middle East respiratory syndrome (MERS) (1), and coronavirus disease 2019 (COVID-19). The COVID-19 outbreak, first identified in Wuhan City, China (2), was caused by severe acute respiratory syndrome coronavirus 2 (SARS-CoV-2), which triggered a global pandemic. Due to its high transmissibility, the virus continues to pose significant health risks (3), as persistent long COVID has been associated with a range of symptoms including headache, fatigue, cognitive-emotional disturbances, pain, cardiopulmonary complications, and sensory alterations such as anosmia and ageusia, which have been reported to last for at least two years post-infection (4).

SARS-CoV-2 is an enveloped virus with a positive-sense, single-stranded RNA genome of approximately 30 kilobases. Several subgenomic RNAs encode a number of accessory proteins as well as four structural proteins: envelope (E), membrane (M), nucleocapsid (N), and spike (S) proteins (5). Among these, the N protein is abundantly expressed during infection and exhibits strong immunogenicity. Therefore, it is frequently used in serological assays and vaccine development efforts (6). Additionally, the N protein is considered an attractive antiviral target and serves as a key marker for viral diagnostics (7,8), owing to its two conserved domains: the N-terminal domain and the C-terminal domain (9,10). Numerous studies have reported the detection of N protein using electrochemical immunosensors and enzyme-linked immunosorbent assays (ELISA) (11). Moreover, several peptide regions within the N protein have been identified in the serum, plasma, or saliva of COVID-19 patients (12). However, antigen-based detection typically relies on the use of antibodies, and the performance of these antibodies may vary depending on the supplier or production method. Consequently, molecular diagnostic methods remain essential, with RT-PCR serving as the sensitive assay for detecting the N protein gene (13). Despite its reliability, RT-PCR lacks the rapid, visual readout that is desirable for point-of-care applications.

Lateral flow assays (LFAs), widely recognized as point-of-care diagnostic tools, have been developed using antibodies, nucleic acids, and aptamers for the detection of various biological targets. During the COVID-19 pandemic, several LFAs targeting the antigenic properties of the SARS-CoV-2 N protein were reported (14,15), and some were later commercialized for detecting antibodies in infected individuals (16). Nonetheless, there remains a critical gap in the development of LFAs for the rapid molecular detection of N protein genes. While a few studies have demonstrated the detection of SARS-CoV-2 N gene using LFAs, these approaches have primarily relied on loop-mediated isothermal amplification (LAMP) or reverse transcriptase LAMP (RT-LAMP) (17). Moreover, the target region of the N gene can vary depending on the primers used, with different subregions (N1, N2, N3) commonly utilized for RT-PCR amplification (18,19). This variation highlights the need for alternative molecular detection assays that can target conserved regions of the N gene reliably. Since LFAs can detect specific gene sequences following amplification, they could be integrated with RNA-generating amplification methods for rapid and visual detection (20,21).

In this study, nucleic acid-based lateral flow assays (NABLFAs) were developed for the molecular recognition of the N protein gene region NIID_2019-nCOV_N. A 100-base pair conserved sequence within the target region was used to prepare the assay.

## Material and Methods

### Materials

All chemicals used were of analytical grade, and ultrapure water was employed for the preparation of solutions. Trisodium citrate and HAuCl_4_·3H_2_O were sourced from Alfa Aesar (Germany). The saline-sodium citrate (SSC) buffer was provided by Multicell (USA). Oligonucleotide sequences, with the desired modifications on the 5′-3′ ends, were obtained from Integrated DNA Technologies (USA). Membrane cards were purchased from Whatman (GE Healthcare, Germany). The size and shape of the synthesized gold nanoparticles (AuNPs) were measured using a transmission electron microscope (TESCAN, Brazil). The maximum absorption peak (λ_max_) of the naked and signal probe-conjugated AuNPs was recorded using an EPOCH2 spectrophotometer (BioTek, USA). The particle size distribution of the conjugate was measured using a Litesizer 500 instrument (Anton Paar GmbH, Austria) based on dynamic and electrophoretic light scattering techniques. The target gene, NIID_2019-nCOV_N, was selected based on GenBank accession number MN908947.3, as it is highly conserved. The sequence was 5′-aaa ttt tgg gga cca gga act aat cag aca agg aac tga tta caa aca ttg gcc gca aat tgc aca att tgc ccc cag cgc ttc agc gtt ctt cgg aat gtc gcg cat tgg cat gga agt cac acc ttc ggg aac gtg gtt gac cta cac agg tgc ca-3′, and the specific region, which is underlined, was used for the hybridization on LFAs. For the sandwich assay, thiol modified oligonucleotide, 5′-att agt tcc tgg tcc cca aaa ttt aaa aaa aaa-3′-SH, was named as signal probe and conjugated on AuNPs. The detection probe, labeled with biotin, 5′-bio aaa aaa gcc aat gtt tgt aat cag ttc ctt-3′, was immobilized on the test line, whereas the complementary sequence of the signal probe, also biotin modified, 5′-aaa ttt tgg gga cca gga act aat ttt-3′-bio, was immobilized on the control line.

### Preparation of colloidal AuNPs

HAuCl_4_·3H_2_O was reduced using sodium citrate for the synthesis of colloidal AuNPs in a clean flask (22). Initially, a 1-mM solution of HAuCl_4_·3H_2_O was prepared and stirred until boiling. The color change to red was then achieved by adding 1% citrate to the solution. After cooling, the synthesized AuNPs were filtered and centrifuged four times at 3000 *g*, 10°C, 15 min to concentrate them, resulting in a preparation referred to as 4X AuNPs. For conjugate formation, signal probes were prepared at concentrations of 2, 4, and 8 µM to determine the optimal probe concentration for stable conjugate formation for long-term use and evaluate the effect of the concentration of oligonucleotide sequences coated onto AuNPs on test performance. Then, they were activated with TCEP for 1 h at room temperature, separately. The solutions were then mixed with 1 mL of 4X AuNPs, incubated overnight for covalent binding, and followed by the addition of PBS to enhance stability, with further overnight incubation. After completion of the reaction, centrifugation was performed at 10,000 *g*, 10°C, 15 min and the pellet was resuspended in phosphate buffer (pH 7.4) containing 0.25% Tween 20, 5% BSA, and 3% sucrose. The AuNPs/signal probe conjugate was washed twice and resuspended in the same buffer before being stored at 4°C.

### Preparation of NABLFAs

Nitrocellulose membrane was used as the capture zone, including both the test and control lines. The other components - sample pad, absorbent pad, and conjugate pad used for preparing the NABLFAs - were manually assembled, as described in our previous study (23). Two types of nitrocellulose membranes with different flow rates (M17 and M12) were tested to compare membrane efficiency. Conjugate pads were loaded with AuNPs/signal probe conjugate and dried at 37°C. PBS or SSC buffers were used as running buffers to ensure efficient flow through the membrane. The test and control zones were prepared using the streptavidin-biotin interaction. For this purpose, streptavidin was initially conjugated to biotin-modified oligonucleotides and then immobilized on the membrane as a thin layer using a micropipette. The synthetic target sequence was applied to the sample pad along with the running buffer in a total volume of 100 µL and strip assays were washed with the same buffer as needed. Strip test experiments were conducted with a minimum of two replicates. The intensity of the color obtained from the NABLFAs was captured using ImageJ software 1X (NIH, USA) to evaluate the limit of detection (LOD). For each experiment, a fixed area was selected for all positive test lines, and the mean intensity was acquired. As a control, an area anywhere on the membrane was selected, and the relative intensity of the test line was calculated in reference to the control intensity. A histogram plot, scaled from 0 to 255, was generated to depict the intensity of the band. A two-tailed Student's *t*-test was conducted to statistically compare the mean intensities of the test lines between groups. All data are reported as means±SD, and statistical significance was determined at a threshold of P<0.05. Assumptions of normality and equal variances were assessed prior to analysis to ensure the appropriateness of the *t*-test.

## Results and Discussion

### Analysis of AuNPs and conjugates

**Figure 1:**
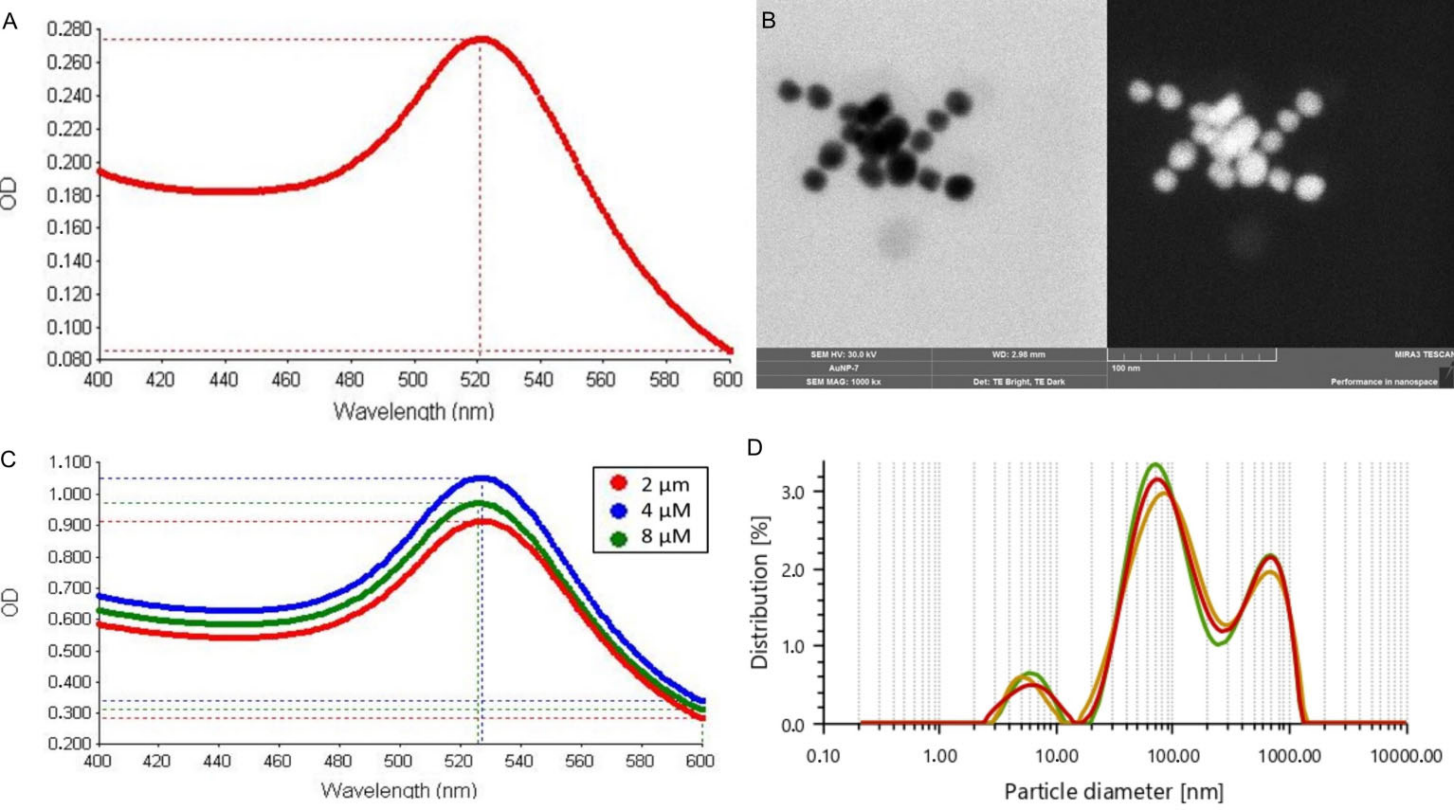
UV-Vis spectroscopy (**A** and **C**) and electron microscopy (**B**) images of the synthesized gold nanoparticles (AuNPs). **A**, Maximum absorption peak of the naked AuNPs, maximum absorption peak (**C**), and particle size measurement (**D**) of the AuNPs/signal probe conjugate.

UV-Vis spectroscopy and electron microscopy were employed to characterize the synthesized AuNPs. The maximum absorbance wavelength (λ_max_) and the average diameter of the unmodified AuNPs were determined to be 521 nm (Figure 1A) and approximately 14 nm, respectively (Figure 1B). The mean size of AuNPs was calculated from the STEM image and found to be 14.30±1.79 nm with the standard deviation 1.79. The concentration of colloidal AuNPs was calculated as 0.4 nM based on the extinction coefficient corresponding to 14 nm particles at the λ_max_ value (24). Following the functionalization of AuNPs surfaces with signal probes, a redshift in λ_max_ was observed from 521 to 528 nm, and the size of the conjugate was 159±4 nm according to the particle size analyzer indicating successful probe conjugation (Figure 1C and D). Although three different probe concentrations (2, 4, and 8 µM) were tested during the conjugation process, no significant differences in color intensity were observed among the resulting conjugates (data not shown), and no aggregation was detected in any of the conjugate solutions. Nevertheless, all probe-conjugated AuNPs were utilized in the preparation of NABLFAs to assess their performance in subsequent analytical steps.

### Detection of target by NABLFAs

**Figure 2:**
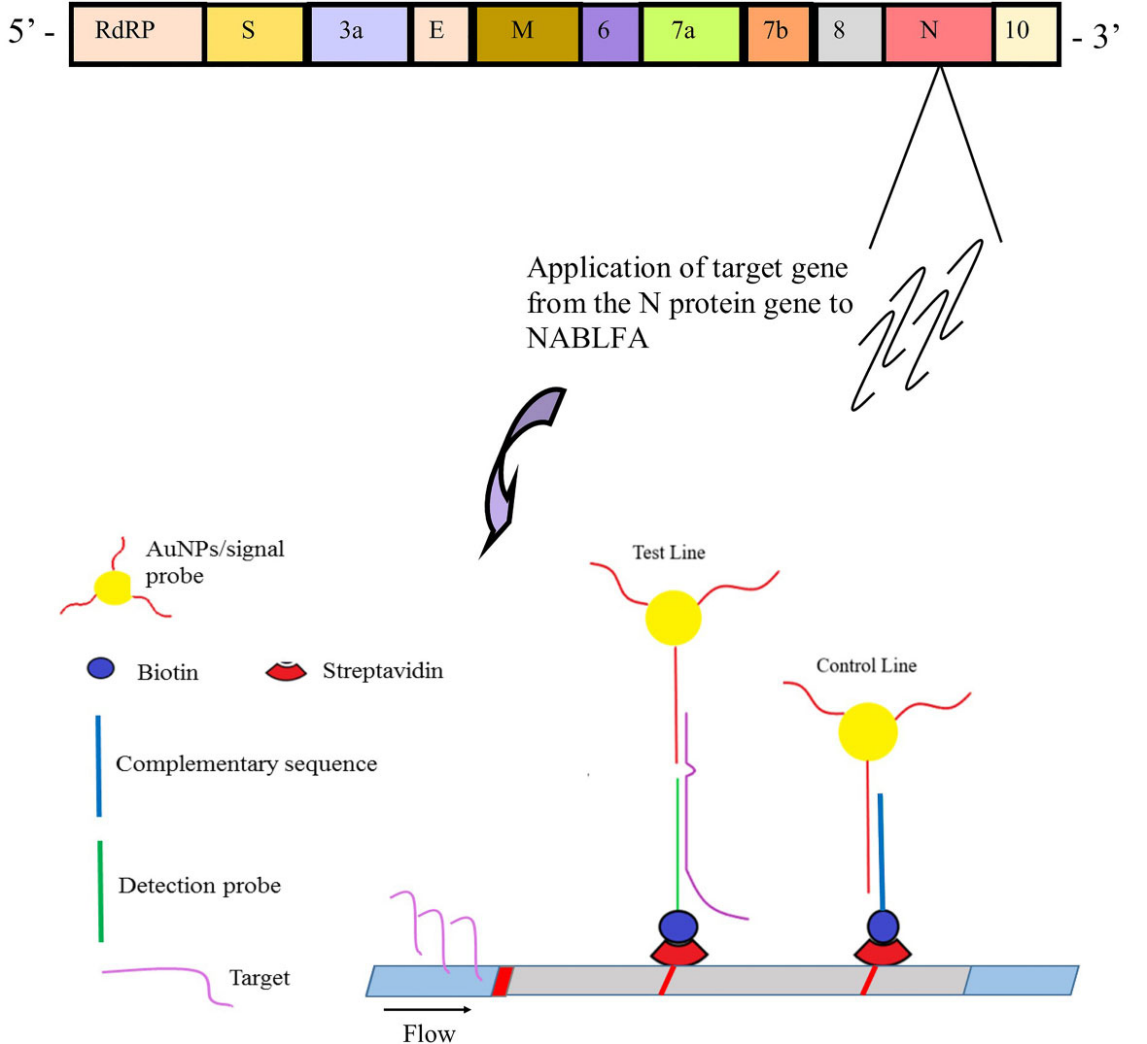
Prepared nucleic acid-based lateral flow assay (NABLFA) and working principle of the assay model for the detection of NIID_2019-nCOV_N region of SARS-CoV-2. The target gene region is loaded onto the sample pad in 100 µL of an appropriate buffer. Due to the flow effect, the target gene region moves to the conjugate pad, where it hybridizes with oligonucleotides conjugated to gold nanoparticles (AuNPs). As the sample continues to flow along the nitrocellulose membrane, the other part of the target gene hybridizes with complementary oligonucleotides on the test line, resulting in the formation of a red color due to the accumulation of AuNPs. Excess conjugation will lead to hybridization with the complementary sequence on the control line, also resulting in a red color formation.

The components of the LFAs were manually assembled, and the working principle of the designed assay is illustrated in Figure 2. Various parameters including signal probe concentrations, LOD, running buffer composition, and two different membrane types were evaluated for the detection of NIID_2019-nCOV_N using the developed strip-based platform.

Based on the results, all of the running buffers tested were found to be suitable for sample migration, and no nonspecific binding was observed on the strip assays regardless of the membrane type or probe concentration used (Supplementary Figure S1). Interestingly, only at the concentration of 8 µM, the target-loaded strip exhibited barely detectable test and control lines, while the negative control functioned as expected (Supplementary Figure S1C-F>). Similarly, no significant differences were noted between the M17 and M12 membranes in terms of assay performance, flow rate, or background signal (Supplementary Figure S1A-F). Given that membrane characteristics play a crucial role in the performance of point-of-care assays, these findings are promising, as they indicate that efficient and selective hybridization occurred on both membranes, independent of other experimental parameters. Therefore, PBS or SSC buffer was selected as the running buffer for subsequent experiments due to their comparable performance across all tested conditions. All three probe concentrations were subsequently employed to assess their influence on the LOD and the specificity of the NABLFAs.

**Figure 3:**
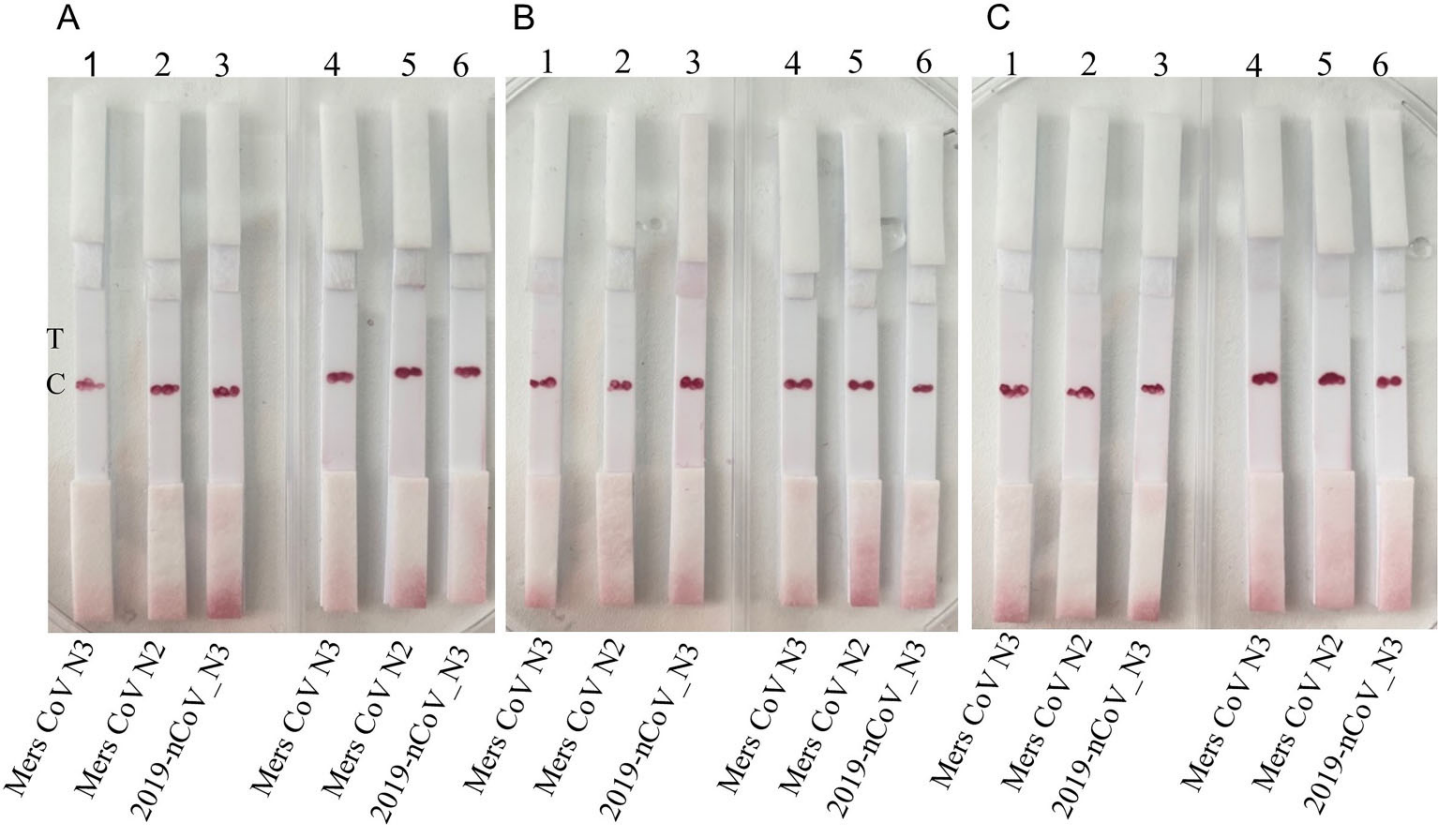
Specificity of the nucleic acid-based lateral flow assay (NABLFAs) developed by M17 (**A1**-**A3**; **B1**-**B3**; **C1**-**C3**) and M12 (**A4**-**A6**; **B4**-**B6**; **C4**-**C6**) membranes using different probe concentrations. **A**, NABLFAs prepared by 2 µM probe. **B**, NABLFAs prepared by 4 µM probe. **C**, NABLFAs prepared by 8 µM. T: Test line; C: Control line.

Initially, the specificity of the prepared assays was evaluated by loading gene fragments specific to MERS-CoV N3, MERS-CoV N2, and 2019-nCoV_N3 onto the NABLFAs. The results demonstrated that the designed strip assays exhibited high specificity toward the target gene, with no false-positive or false-negative signals observed on the test lines across both membrane types and all probe concentrations (Figure 3A-C). Although the 2019-nCoV_N3 gene is located within the N protein gene, the conserved region of the NIID_2019-nCoV_N gene, which is located in another region of the N protein gene, did not show nonspecific binding among the negative controls. This indicates that the designed probes possess strong hybridization efficiency with the target gene through a sandwich hybridization mechanism, and that nonspecific interactions were absent in the absence of the target sequence. Therefore, the assay was found to be a promising candidate for simple, molecular-level detection of SARS-CoV-2, without the need for additional labeling or complex instrumentation offering a distinct advantage over other NABLFA strategies developed for specific detection of the N gene (25-27). Furthermore, the assay design employed here offers a more practical approach than dual LFA systems, which require biotinylated PCR products and antibody-based recognition for nucleic acid detection (28,29). It is also noteworthy that the presence of polyA tails on both the signal and detection probes did not interfere with target capture, in contrast to some RNA detection strategies that rely on adjacent sequence hybridization in LFAs (30).

**Figure 4:**
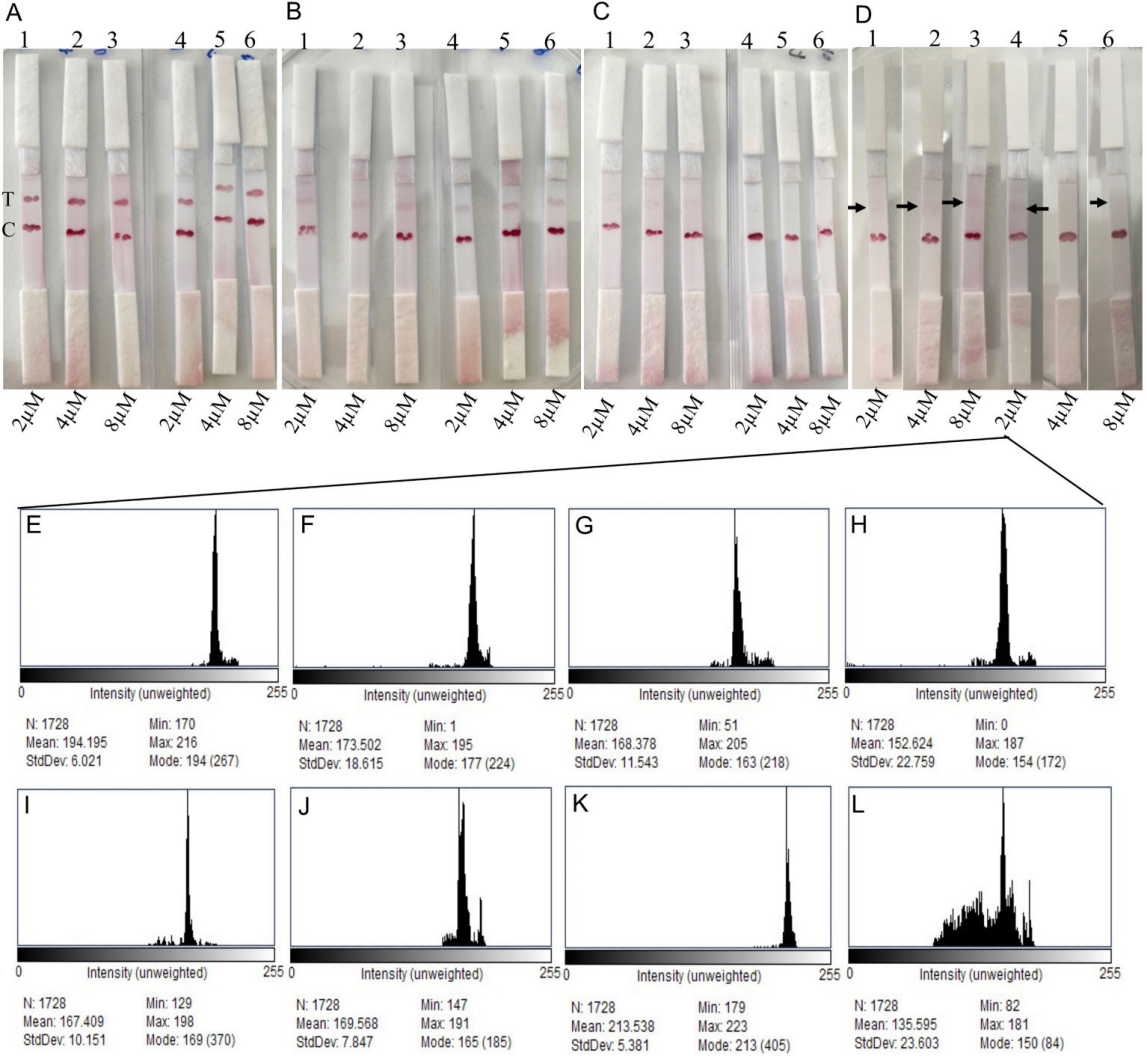
Limit of detection (LOD) of the nucleic acid-based lateral flow assay (NABLFA) by M17 (**A1**-**A3**; **B1**-**B3**; **C1**-**C3**; **D1**-**D3**) and M12 (**A4**-**A6**; **B4**-**B6**; **C4**-**C6**; **D1**-**D3**) using different probe concentrations. Histogram plots of the strips obtained from the D image (**E**-**L**). **A**, 0.01 µM target. **B**, 1 nM target. **C**, 0.1 nM target. **D**, 1 pM target. **E**-**J**, Histogram plot of the test lines of **D1**-**D6**, respectively. **K**, Histogram plot of the empty area on the strip**. L**, Histogram plot derived from the control line of the strip **D4**. Arrows show the test lines. T: Test line; C: Control line. Data are reported as means±SD. Statistical comparisons of test line intensities between groups were performed using a two-tailed Student's *t*-test. Differences were considered statistically significant at P<0.05, with the results demonstrating significance (P=0.015).

To determine the LOD of the NABLFAs, the target sequence was applied to the assay at concentrations of 0.01 µM, 1 nM, 0.1 nM, and 1 pM. As shown in Figure 4, the LOD was identified as 1 pM on the strips developed with both membrane types (Figure 4D). This detection limit was notably lower than that reported for DNA-functionalized microcantilever sensors used for detecting alternative N gene regions of SARS-CoV-2 (31) and also compared favorably with highly sensitive electrochemical DNA sensors (32-34). Although 1 pM was consistently detected across all probe concentrations and both membrane types (Figure 4D), the test line on strip D5 showed weak or unclear signal (Figure 4D5), which may be attributed to suboptimal interactions between the capture reagent and the target, as previously reported (23). It should be noted that while the 0.1 nM target was detectable on the M17 membrane (Figure 4C1-C3), detection on the M12 membrane was only achieved weakly when using 2 µM probe concentration (Figure 4C4), with line intensity comparable to that observed at 1 pM detection (Figure 4D4). Interestingly, while 0.1 nM of the target could not be detected using the M12 membrane and a conjugate prepared with 8 µM of probe (Figure 4C6), a faint but measurable signal was obtained for 1 pM of the target under the same conditions. This phenomenon may be attributed to the influence of target concentration on the interaction between the conjugate and the target, which can in turn affect the efficiency of sandwich complex formation. Additionally, the electrostatic properties of the interacting DNA molecules may play a critical role in detection, as their charge characteristics can significantly impact binding dynamics and signal generation (35). Thus, these observations highlight the importance of optimizing probe concentration, target load, and membrane selection to achieve reliable and sensitive detection in LFA systems.

In addition to visual detection, LOD was confirmed through histogram analysis based on test line intensities. To assess the color development and signal strength, histogram plots were generated from the strips shown in Figure 4E-L. On strips D1-D4 and D6, the test line signals were weakly visible to the naked eye and supported by histogram analysis (Figure 4E-J), with results showing statistical significance (P=0.015). Moreover, histogram plots generated from the control lines and blank regions on the strips clearly demonstrated intensity differences (Figure 4K-L). These findings highlight that using approximately 100-fold lower concentrations of capture probes renders the NABLFAs cost-effective compared to conventional nucleic acid detection methods that require higher probe concentrations (30).

Additionally, the results demonstrated that the hybridization efficiency at the control line remained consistent across NABLFAs prepared using all three probe concentrations, as indicated by comparable line intensities (Figure 4A-D). This suggests that a 2-µM probe concentration was sufficient for the development of NABLFAs targeting the specific gene sequence, given that no significant differences in performance were observed among the different probe conjugates. As expected, the test line signal weakened with decreasing target concentration due to reduced hybridization efficiency associated with the lower availability of the target gene (Figure 4A-D). Moreover, the results confirmed that the selected probe concentrations, both on the AuNPs surfaces and within the capture zones, were optimal for achieving sensitive hybridization. Thus, the designed NABLFAs enabled rapid, visual, and sensitive molecular detection of SARS-CoV-2 without the need for advanced instrumentation or labeling strategies. When compared to antigen- or antibody-based LFAs, as well as LAMP-based diagnostic assays for SARS-CoV-2, the present approach offers a promising alternative for direct molecular detection of the viral genome. In addition, NABLFAs are advantageous as they provide molecular diagnostics, facilitate clinical decision-making, and require minimal sample volume. However, as indicated in our study, they may require optimization of several parameters, as molecular tests based on hybridization can lead to false-negative or false-positive results. Additionally, NABLFAs may require amplification for clinical samples and well-trained personnel, thus these exceptional conditions should be considered in the development of future NABLFAs.

In conclusion, the molecular detection of the NIID_2019-nCOV_N gene region specific to SARS-CoV-2 was successfully achieved by developing NABLFAs with a low LOD. The results demonstrated that both the buffers and signal probes immobilized on AuNPs play a critical role in enabling efficient hybridization without nonspecific binding. To the best of our knowledge, this is the first study to report the recognition of the NIID_2019-nCOV_N gene region by NABLFAs for rapid detection, without the need for advanced experimental procedures or instrumentation. Furthermore, the findings suggest that the designed assay model has the potential to be adapted for both clinical samples and mutant strains of the virus. Therefore, we plan to further develop NABLFAs for the rapid detection of this gene region using clinical samples from SARS-CoV-2 infected patients, allowing easy preparation, fast results, and visual diagnosis for improved human health surveillance.

## Supplementary Materials

Supplementary Material Click here to view [pdf].

## Data Availability

All data generated or analyzed during this study are included in this published article.
