# Supplementary material for: Design of a lateral flow assay targeting the conserved NIID_2019-nCoV_N gene region for molecular viral diagnosis
Source: Braz J Med Biol Res. 2025 Oct 13;58:e14761. doi: 10.1590/1414-431X2025e14761 (PMC12519558; doi:10.1590/1414-431X2025e14761)

Figure S1. Evaluation of nucleic acid-based lateral flow assay (NABLFA) performance using 2 μM (**A** and **D**), 4 μM (**B** and **E**), and 8 μM (**C** and **F**) probe concentrations on different membrane types in combination with various running buffers. **A**–**C**, M17 membrane. **D**–**F**, M12 membrane. T: Test line; C: Control line.

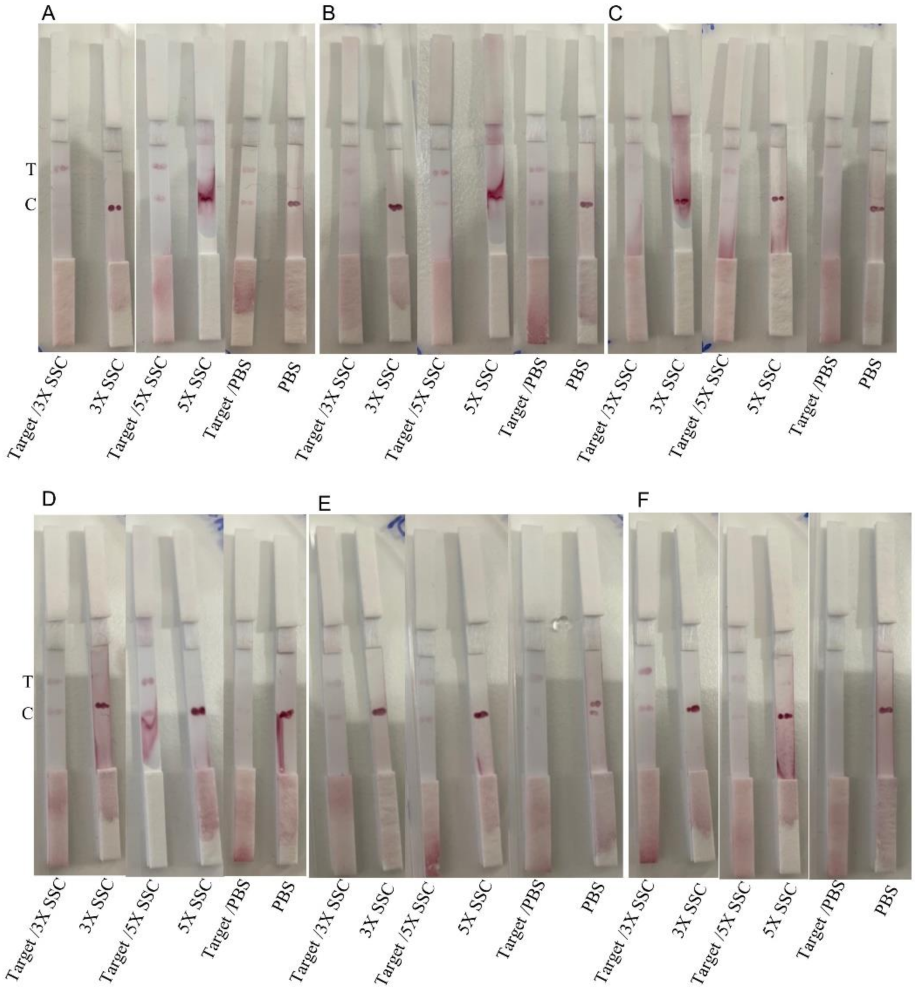

Supplement: Supplementary file 1 [file 1414-431X-bjmbr-58-e14761-suppl.pdf]
